# Meta-Analysis of Randomized Controlled Trials of Xueshuantong Injection in Prevention of Deep Venous Thrombosis of Lower Extremity after Orthopedic Surgery

**DOI:** 10.1155/2020/8877791

**Published:** 2020-11-27

**Authors:** Shu-ting Yan, Feng Gao, Tai-wei Dong, Hao Fan, Miao-miao Xi, Feng Miao, Pei-feng Wei

**Affiliations:** ^1^College of Pharmacy, Shaanxi University of Chinese Medicine, Shiji Road, Qindu District, Xianyang, Shaanxi 712046, China; ^2^The Second Affiliated Hospital of Shaanxi University of Traditional Chinese Medicine, Weiyang West Road, Qindu District, Xianyang, Shaanxi 712046, China

## Abstract

**Objective:**

To systematically evaluate the clinical efficacy of Xueshuantong injection (*Panax notoginseng* saponins) in preventing deep venous thrombosis (DVT) of lower extremity after orthopedic surgery.

**Methods:**

The randomized controlled trials (RCTs) of Xueshuantong injection in prevention of lower extremity DVT after orthopedic surgery were retrieved from CNKI, Wanfang database, VIP, PubMed, and Cochrane Library by August 2020. Revman5.2 was used to analyze the results.

**Results:**

A total of 20 articles including 2336 patients were included. The results of meta-analysis showed that the incidence of DVT in the experimental group was lower than that in the control group; after operation, the D-dimer (Ddimer), thrombin time (APTT), and prothrombin time (PT) in the experimental group were significantly improved compared with those in the control group, and the difference between the two groups was statistically significant.

**Conclusion:**

Xueshuantong injection can effectively prevent the formation of lower extremity DVT after orthopedic surgery and antagonize the postoperative hypercoagulable state of blood, which has high clinical value.

## 1. Background

DVT is a common perioperative complication of fractures, with a potential disability rate and fatality rate of 40%∼70% for patients with traumatic fractures [1–4]. If venous thrombosis is not treated in time, it may cause swelling, pain, and dysfunction of affected limbs in mild cases, and in severe cases, pulmonary embolism will occur and even life-threatening when thrombus enters the pulmonary circulation [5–9]. According to current research reports, the incidence of DVT after fracture surgery is as high as 9%∼62%. The Guidelines of the American College of Chest Physicians (ACCP) and the American College of Orthopedic Surgeons (AAOS) all suggest that if there are no contraindications, the drugs or physical methods should be used to prevent venous thromboembolism after fracture surgery [10, 11]. Xueshuantong injection (*Panax notoginseng* saponins) has antiplatelet aggregation effect and is widely used in orthopedic surgery for thromboprophylaxis [12, 13]. At present, the clinical studies on Xueshuantong injection to prevent venous thrombosis of lower extremity after fracture surgery are mainly concentrated in China. In contrast, there is a lack of meta-analysis on the prevention of DVT after fracture with Xueshuantong injection. Therefore, the purpose of this meta-analysis is to examine the efficacy of Xueshuantong injection in the prevention of DVT by summarizing the existing clinical studies.

## 2. Data and Methods

### 2.1. Retrieval Strategy

According to the PRISMA, two researchers independently searched PubMed, Cochrane Library, CNKI, Wanfang, and VIP. To conduct a comprehensive search, studies published prior to August 15, 2020, were investigated without language limitations. The search terms used were as follows: “Xueshuantong” and “fracture” and “deep venous thrombosis of lower” or “DVT.” All corresponding articles were downloaded into NoteExpress (version 3.0, Beijing, China) for further investigation.

### 2.2. Inclusion Criteria

According to the guidelines for the prevention of venous thromboembolism in Chinese orthopedic surgery, color Doppler ultrasound examination has gradually superseded venography as the primary diagnostic procedure, which is a preferred method for the diagnosis of DVT with high sensitivity and accuracy. DVT screening was required for all patients before and after treatment. (1) Study type: randomized controlled trial, blinded or not, complete data, and language limited to Chinese/English. (2) Subjects: patients with fractures confirmed by imaging examination and requiring surgical treatment. (3) Intervention measures: Xueshuantong injection or Xueshuantong injection combined with other drugs was used in the experimental group, while low molecular weight heparin sodium (LMWH), other anticoagulants, or blank control were used in the control group. (4) The following indices in the articles must contain at least one of the following: incidence of DVT, D-D, PT, and APTT.

### 2.3. Exclusion Criteria

(1) Non-RCT study. (2) Repetitive articles. (3) Nonoriginal research. (4) Patients with DVT before the surgery, included patients with conditions that may easily cause DVT. (5) Studies such as reviews, animal experiments, and case reports that were considered to be irrelevant to the theme.

### 2.4. Data Extraction

Two researchers (Shu-ting Yan and Feng Gao) independently searched and extracted the data. When the opinions were different, they discussed together or asked another author for advice (Tai-wei Dong). This information is provided and arranged in Table 1.

### 2.5. Literature Quality Evaluation

Two researchers independently evaluated each of the included articles according to the bias risk assessment tool in the Cochrane Handbook. Evaluation contents: (1) whether the random sequence is generated properly. (2) Whether the random distribution is hidden. (3) Whether the blind method is used. (4) Whether the result data are reported completely. (5) Whether there is selective report. (6) Whether there is other bias.

### 2.6. Statistical Analysis

The analysis was performed by RevMan5.2 software. *I*^2^ test (*p*=0.1) was used for heterogeneity analysis between included study results. *I*^2^ < 50% indicated no statistical significance for heterogeneity among studies, and the fixed effect model was used for heterogeneity analysis; *I*^2^ ≥ 50% showed that the heterogeneity among the studies was statistically significant, and random effect model analysis was used. In the continuous variable study, the weighted average difference (WMD) was used as the effect indicator, and the risk ratio (RR) was used as the effect indicator of the dichotomous variable. For each study, we calculated the risk ratios (RRs) with their 95% confidence interval (CI), and *p* < 0.05 indicated a statistically significant difference. Begg's test was used to assess publication bias using STATA 13.0 statistical software (Stata Corp, College Station, TX, USA), and *p* < 0.1 indicated a statistically significant difference.

GRADE profiler software was used to input and quantify the quality of evidence for the included outcome indicators.

## 3. Result

### 3.1. Results of Study Retrieval

**Figure 1:**
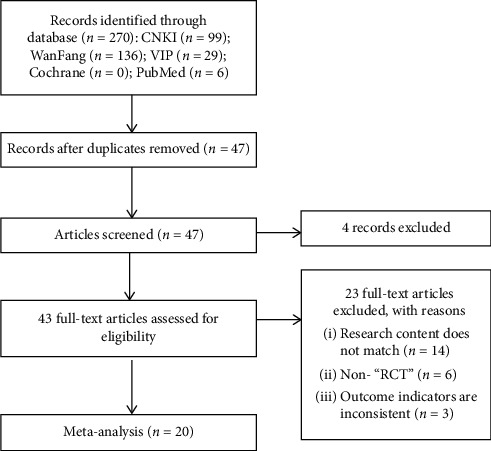
Document screening flowchart.

Studies took place between 2006 and 2019 (Table 1). A total of 270 potentially corresponding studies were identified by our primary search, and 223 articles were exempted for repeat. Then, a full-text review was conducted on the remaining 47 articles. A total of 27 studies were exempted for the following reasons: 18 articles had vague diagnoses, 6 articles are not RCTs, and the results of the 3 articles are inconsistent. Twenty studies had adequate index data to permit the calculation of effect sizes for inclusion in this meta-analysis (Figure 1). Of the 20 included studies, 2336 patients with fractures underwent fracture surgery (1175 cases in the experimental group and 1161 cases in the control group) and used in this meta-analysis.

### 3.2. Literature Quality Evaluation

**Figure 2:**
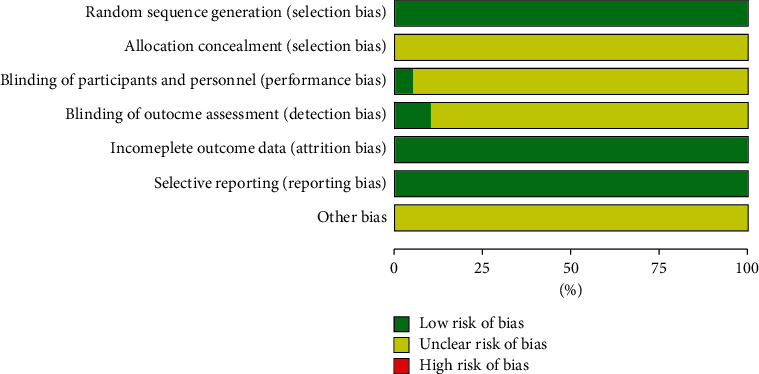
Document quality evaluation chart.

All trials were RCTs of participants according to Cochrane risk of bias estimation. Particular information on distribution was absent from most articles. All studies did not use blinding of participants and consequence assessment. All articles had integral outcome data with a low risk of attrition bias and low risk of reporting bias as detailed results are given (Figure 2).

### 3.3. Meta-Analysis Results

#### 3.3.1. Comparison of DVT Incidence

**Figure 3:**
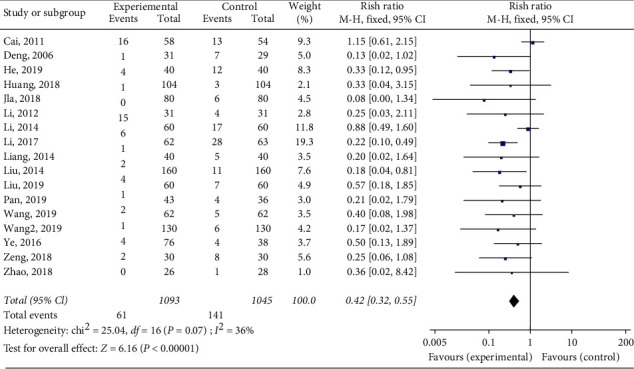
Forest map of incidence rate of DVT after Xueshuantong injection for preventing fracture.

A total of 17 articles reported the incidence of DVT. The fixed effect model was used for analysis. The results showed that the incidence of DVT after fracture surgery could be significantly reduced by using Xueshuantong injection (RR = 0.42, [95% CI (0.32, 0.55)], *p* < 0.00001; Figure 3). There was no statistically significant heterogeneity among the individual trials (*I*^2^ = 36%, *p*=0.07).

#### 3.3.2. D-D

**Figure 4:**
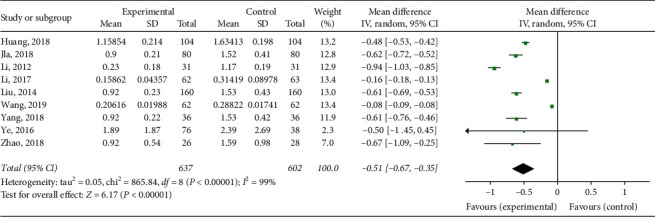
Forest map of D-D level in patients with Xueshuantong injection after fracture prevention.

10 studies measured D-D levels in patients after fracture surgery. There was a statistically significant degree of heterogeneity among individual studies (*I*^2^ = 99%, *p* < 0.00001); therefore, a random effect model was performed for a meta-analysis, which showed that Xueshuantong injection or it combined with other treatment can reduce patients' D-D level (MD = −0.51 [95% CI (−0.67.88, −0.35)]; Figure 4).

#### 3.3.3. PT

**Figure 5:**
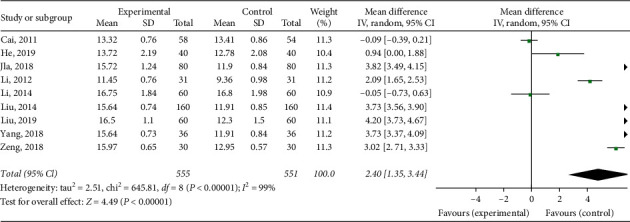
Forest map of PT level in patients with Xueshuantong injection after fracture prevention.

Nine studies observed PT levels in patients after fracture surgery. There was a statistically significant degree of heterogeneity among individual studies (*I*^2^ = 99%, *p* < 0.00001); therefore, a random effect model was performed for a meta-analysis, which showed that Xueshuantong injection or it combined with other treatment can improve patients' PT level (MD = 2.40 [95% CI (1.35, 3.44)]; Figure 5).

#### 3.3.4. APTT

**Figure 6:**
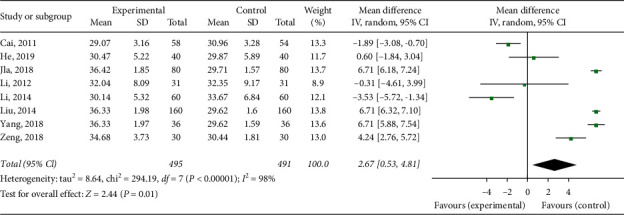
Forest map of APTT level in patients with Xueshuantong injection after fracture prevention.

A total of 9 articles measured APTT. There was a statistically significant degree of heterogeneity among individual studies (*I*^2^ = 98%, *p* < 0.00001); therefore, a random effect model was performed for a meta-analysis, which showed that Xueshuantong injection or it combined with other treatment can improve patients' APTT level (MD = 2.67 [95% CI (0.53, 4.81)]; Figure 6).

#### 3.3.5. Publication Bias Was Assessed Based on the Incidence of DVT

**Figure 7:**
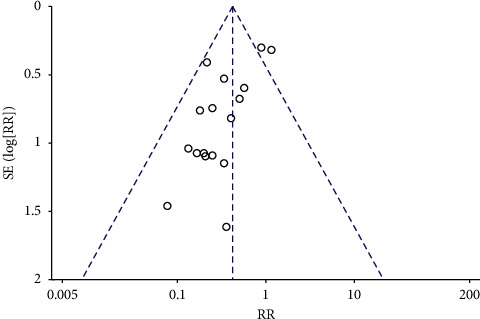
Funnel plot of incidence rate of DVT after Xueshuantong injection for preventing fracture.

**Figure 8:**
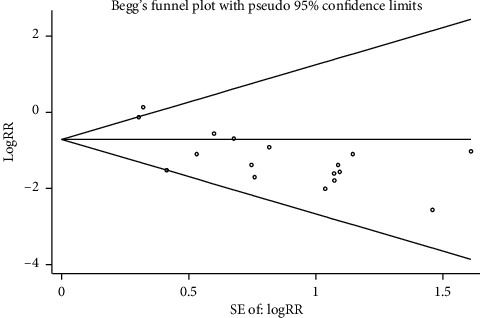
Begg's regression diagram with incidence of DVT.

The incidence of DVT included in the literature was evaluated (Figure 7). Funnel plot results indicate that there is a certain publication bias in the study of Xueshuantong injection in the prevention of lower extremity deep vein thrombosis after orthopedic surgery. In order to further evaluate whether there is publication bias, STATA 13.0 software was used for the Begg test, and the results showed that *Z* = 0.87, *p*=0.387, in which *p* < 0.05, had statistical difference, indicating no publication bias (Figure 8).

#### 3.3.6. Sensitivity Analysis

The sensitivity analysis was carried out for the studies with *I*^2^ > 50%. After each study was excluded one by one, the systematic evaluation was conducted. The change of *I*^2^ was not significant, and the results did not change, indicating that the systematic evaluation was stable, and the results were reliable.

#### 3.3.7. Grading Evaluation of Evidence Quality

**Figure 9:**
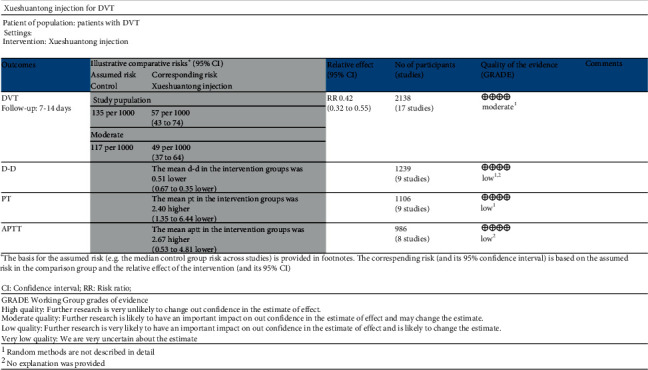
Grading evaluation chart of evidence quality.

GRADE was used to grade the evidence quality of the included literature. The incidence of DVT was the key outcome indicator, while D-D, PT, and APTT were the important outcome indicators. The results showed that the incidence of DVT was of moderate quality, and the other three indexes were all of low quality (Figure 9).

## 4. Discussion

The results of meta-analysis showed that the incidence of DVT and the level of D-D, PT, and APTT in the experimental group were better than those in the control group. The difference between the two groups was statistically significant, suggesting that Xueshuantong injection has played a great advantage in the prevention of deep venous thrombosis of lower limbs.

The incidence rate of DVT is caused by congenital factors in the postoperative stage of fracture, such as surgical correction, infection, and activity level [34, 35], especially in large department of orthopedics operations, such as total hip replacement and total knee replacement, the risk of hip fracture is the largest [36, 37]. For patients with traumatic fracture, huge external energy such as falling injury or traffic injury may lead to vascular damage. In addition, immobility combined with long-term bed rest will slow down venous return, making patients prone to DVT, which may lead to lower limb paralysis or even death [38, 39]. Although low molecular weight heparin calcium, rivaroxaban, and other anticoagulants are widely used in patients with traumatic fracture, the incidence of perioperative DVT is still very high, so there are great challenges in exploring the prevention of postoperative DVT in fracture patients [40–42]. Traditional Chinese medicine (TCM) classifies DVT into the categories of “blood stasis syndrome,” “femoral swelling,” “pulse obstruction,” and “edema.” The main pathological factors are blood stasis. Due to surgical trauma and bed rest, the blood is damaged, the vein is damaged, the blood does not pass through, the blood away from the pulse is blocked, and the reflux is not smooth, which leads to thrombosis. Blood stasis can cause skin temperature to rise and become painful for a long time. According to the patient's constitution, kidney tonifying, heat clearing, dampness removing, and other methods are used. Xueshuantong injection is a traditional Chinese medicine injection made of total saponins extracted from *Panax notoginseng*, which belongs to a modern dosage form of Chinese patent medicine [43]. Modern research shows that the main effective component of Xueshuantong injection is *Panax notoginseng* saponins, which can be widely used to treat various diseases, such as atherosclerosis, acute lung injury, cancer, and cardiovascular diseases [44–46]. In the related basic research and clinical application, its exact curative effect has been confirmed.

From the results of this study, the use of Xueshuantong injection in the prevention of lower limb venous thrombosis after fracture surgery was significantly lower than that of the control group, worthy of further study. In this meta-analysis, we mainly studied the efficacy of Xueshuantong injection to prevent the incidence of DVT in patients after fracture surgery in China and made a systematic analysis. On the basis of comparing the incidence of DVT, we also included the commonly used clinical observation indexes such as D-D, PT, and APTT, so as to observe the effect of Xueshuantong injection more comprehensively. However, there are still some deficiencies in this meta-analysis: (1) Although all the included literatures mentioned the method of randomized grouping, some of them lacked a detailed description of the concealment of the randomized scheme; (2) the number of included literatures was small, all of them were Chinese literatures, and there were some problems such as large sample size gap between literatures, insufficient detailed basic data, and incomplete unification of treatment cycle; (3) all the included literatures were published articles. If there are no unpublished results, studies with negative results may be missed, and there is a risk of reducing the strength of the argument. Therefore, the evidence strength of the conclusion of this study needs to be further improved, and more high-quality RCTs are needed to verify, so as to obtain a more accurate conclusion on the effect of Xueshuantong injection in preventing lower extremity deep vein thrombosis after fracture surgery, so as to provide good clinical guidance for the prevention of lower extremity deep vein thrombosis after fracture surgery.

## Figures and Tables

**Table 1:** Principal characteristics of the studies included in the meta-analysis.

No.	Included study	Sample size (T/C)	Experimental group		Control group		Treatment course (days)	Evaluation indicators
			Treatment	Age (years)	Treatment	Age (years)		
1	Cai [14]	58/54	Xueshuantong injection	56.6	LMWH	56.6	14	①④⑤
2	Deng [15]	31/29	Xueshuantong injection	44 ± 1.6		45 ± 1.5	7	①
3	He and Cao [16]	40/40	Xueshuantong injection + LMHC	62.87 ± 1.65	LMHC	62.45 ± 1.28	14	①④⑤
4	Huang[17]	104/104	Xueshuantong injection + Tongmaidan	67	LMHC	68	7	①②
5	Jia and Tang [18]	80/80	Xueshuantong injection	84.30 ± 1.61	Conventional treatment	84.21 ± 1.58	7	①②④⑤⑥
6	Li [19]	31/31	Xueshuantong injection	52.2 ± 8.9	Conventional treatment	50.5 ± 10.6	6	①②④
7	Li et al. (2014) [20]	60/60	Xueshuantong injection	57.93 ± 2.68	LMWH	58.79 ± 2.97	14	①④⑤
8	Li [21]	62/63	Xueshuantong injection + rivaroxaban	35.1 ± 9.1	Xueshuantong injection	34.7 ± 8.9	14	①②⑥
9	Huang [22]	40/40	Xueshuantong injection + LMHC	59.3 ± 3.3	LMHC	60.0 ± 2.9	7	①
10	Liu [23]	40/40	Xueshuantong injection		LMHC		14	⑦
11	Liu [24]	160/160	Xueshuantong injection	76.5 ± 5.3	Conventional treatment	76.5 ± 5.3	7	①②④⑤⑥
12	Liu [25]	62/62	Xueshuantong injection	65.91 ± 12.25	Rivaroxaban	65.89 ± 12.22	14	①②⑥
13	Pan and Tang[26]	43/36	Xueshuantong injection	52.6 ± 1.1	LMHC	51.8 ± 1.5	14	①⑥
14	Wang [27]	62/62	Xueshuantong injection	71.69 ± 5.02	Rivaroxaban	72.16 ± 6.21	14	①②⑥
15	Wang [28]	130/130	Xueshuantong injection	46.62 ± 12.15	Conventional treatment	46.15 ± 12.12	3	①③
16	Wu [29]	40/40	Xueshuantong injection	73.45	LMHC	70.58	14	③
17	Yang [30]	36/36	Xueshuantong injection + low-frequency physiotherapy	46.5 ± 5.3	Conventional treatment	45.4 ± 4.1	14	②④⑤
18	Ye et al. ([31]	38/38	Xueshuantong injection + electric acupuncture	68 ± 5	LMHC	66 ± 3.4	14	①②③
19	Zeng et al. ([32]	30/30	Xueshuantong injection	61.23 ± 4.82	LMHC	61.34 ± 4.34	14	①②④⑤⑥
20	Zhao ([33]	28/26	Xueshuantong injection + LMHC	45.62 ± 18.89	LMHC	45.20 ± 15.48	10	①②③⑥

*Note*. ①: DVT; ②: D-D; ③: therapeutic effect; ④: PT; ⑤: APTT; ⑥: hemorheology; ⑦: curative effect.

## Data Availability

The data used to support the findings of this study are included within the article.
